# Edward Livingston Trudeau, M.D., and R. L. Stevenson: Fellow-Sufferers from Tuberculosis

**Published:** 1916-05-06

**Authors:** 


### EDWARD LIVINGSTON TRUDEAU, M.D., and R. L. STEVENSON.

### Fellow-sufferers from Tuberculosis.

Edward Livingston Trudeau, M.D., who died last year, and whose "Autobiography" has just been published in America (Doubleday, Page and Co., New York), as well as another volume about him called "The Beloved Physician," by Stephen Chalmers, was one of the most heroic figures in recent medical science. It was quite characteristic of him that his favourite statue, a parable of his own life, was "Gloria Victis," by Mercié. This fine work was inspired in 1871, after the French had been crushed by Germany. The piece represents a young gladiator who has just received his death-blow while facing the foe. In falling, with his broken sword in his hand, he is caught by the figure of Victory, with outstretched wings, who swoops down and bears him aloft in her arms. When this young New York physician was stricken with consumption soon after his marriage, he did not lose heart. He found health again round Saranac Lake, in the Adirondack Mountains, then but a resort of hunters and fishers. He had a dream to make this place a health resort for others, which he made a reality. After he found health in hunting and fishing among the pine woods and by the lakes of the Adirondacks, denied to him in New York, he resided there with his young family. He founded at Saranac, on the northern border of New York State, close to Canada, the Adirondack Cottage Sanatorium for the treatment of incipient consumption in working men and women, the first of its kind in America. Later he founded the Saranac Laboratory for the study of tuberculosis, also the first research laboratory of its kind in the United States. In the forty years of his residence there thousands gratefully acknowledged that their lives had been lengthened through his agency. An interesting invalid came thither in the fall of 1887, R. L. Stevenson, with his wife, mother, stepson, and other members of his household. There he also found that measure of health which allowed him to accomplish the yacht voyage to the South Seas after he left in April 1888. And he wrote there the greater part of the "Master of Ballantrae," and some fine essays for *Scribner's Magazine*. A tablet showing R. L. Stevenson in three-quarter length has been placed on the cottage where he resided. He had faced the severity of the weather in an Astrakhan cap, fur coat, and Indian boots. The tablet has this inscription: "Here dwelt Robert Louis Stevenson during the winter of 1887-1888."

### A Notable Personality.

Edward Livingston Trudeau, born at New York October 5, 1848, came of a marked medical ancestry on both sides of the house. He inherited a passion for wild places and spaces which reconciled him to life in the Adirondacks. His father was a member of a well-known New Orleans family; his mother's father, Dr. François Berger, was a French physician, whose ancestors were physicians for many generations, as far back as could be traced. Young Trudeau spent several years in France with his mother, and returned to the States at the conclusion of the Civil War. He might have succumbed to the temptations and dissipations of New York life and never graduated, but for the influence upon him of a brother whom he nursed until he died of consumption, and of Miss Beare, the daughter of a clergyman, to whom he was married June 29, 1871. They took a honeymoon journey to Europe. He began practice in New York in 1872, and bid fair to build up a good business when stricken with tuberculosis. He had tried many things as a youth, but found his groove in medicine, and now this blow seemed the end of all things. The idea of his sanatorium was obtained from reading in Anstie's "English Practitioner" an account of a visit to Brehmer's Sanatorium in Silesia. He improved on his model, founded his working-class establishment for those who could not pay the high prices asked in the houses which had been built for invalids at Saranac, and Dr. Koch's discoveries encouraged him in the foundation of his laboratory. People came to Saranac from all parts of the States, and found new health and hope in contact with the keen air of that mountain region, and the skill of the now famous doctor and his assistants. Dr. Osler mentioned with approval the work here, in his "Practice of Medicine," 1893.

### Stevenson at Saranac.

It was in Andrew Baker's cottage on the outskirts of the village of Saranac that Stevenson spent the fall and winter of 1887-1888. It was with him a period of great literary activity after he settled down with his wife, mother, and other members of his household. Here he wrote, as we have said, most of the "Master of Ballantrae," and the series of papers for *Scribner's Magazine* which now form a portion of his published volumes. Here also awoke the desire for a yacht, and a cruise in the Pacific which materialised immediately afterwards. Dr. Trudeau tells us that Stevenson was not really ill while here, and benefited more than any of his family, so that he had comparatively few professional calls to make upon him. "He was," says the doctor, "so attractive, however, in conversation, that I found myself, as it was growing dark, very often seated by the big fireplace in the Baker cottage having a good talk with my illustrious patient. Mr. Stevenson was very democratic in his ideas, simple in his mode of life, and disliked dress parade entertainments and the restraints and glitter of society etiquette." This was shown in what he said to the doctor when at dinner with a stylish New York family, with lighted candles, flowers, glassware, and silver. "This sort of thing always gives me stage-fright: does it affect you that way?" Saturday was Stevenson's day for receiving visitors, but one day, when friends knocked at the front door, a voice from within called out, "You cannot come in this way: the wood is up against the door. Go round by the kitchen." On that occasion Mr. Lloyd Osbourne endeavoured to get tea for the ladies, but returned saying, with a broad smile on his face, "I am sorry, you cannot have any tea; the cook scouts the idea." When Stevenson closed the door behind one enthusiastic lady who had bored him, he said with emphasis, "Trudeau, it is not the great unwashed whom I dread; it is the great washed." The doctor was keenly aware of the great privilege he had in meeting Stevenson, and appreciated his "striking personality, his keen insight into life, his wondrous idealism, his nimble intellect, and his inimitable vocabulary in conversation." He said to Trudeau in his laboratory, "Trudeau, your light may be very bright to you, but to me it smells of oil like the Devil." The doctor explains this away by saying that he lived in a world of facts, and Stevenson in an ideal world of fancy. Stevenson, on leaving, showed his appreciation of Dr. Trudeau by presenting him with a unique series of his published works up till that period, with characteristic inscriptions in his own handwriting to each member of the doctor's household, not omitting the dog Nigg. Unfortunately this fine set perished in a fire which consumed his house and laboratory in 1893.

The Laennec Society of the Johns Hopkins Hospital has lately held a special memorial meeting in honour of Dr. Trudeau, at which three physicians, who have been closely associated with him, spoke of his work, his worth, and his character. He was an ardent hunter and horseman, as well as a tireless worker in the ward and the laboratory. Commercial success he utterly despised; and he would have no share in, or profit from, the commercial exploitation of Saranac Lake which followed the fame of his doings there. He had the secret of securing the absolute confidence and the implicit obedience of his patients: needless to say, he was a strict disciplinarian, by inspiring faith not fear. His energy and enthusiasm for tuberculosis research were qualities he seemed able to impart to others; and, indeed, an eye for capacity in his juniors was one of his salient characteristics. A sort of instinct for and attraction to a young man of promise remained with him to the end of his days; and young medical men suffering from tuberculosis seem to have often been his favourite patients.

His knowledge of human nature, his humour, his kindliness and sympathy led to his being often consulted by ex-patients and others on affairs non-medical altogether. He was adviser and father-confessor to scores of perplexed patients of both sexes; and he kept up a correspondence with hundreds of those who had stayed at Saranac under his care. Withal Trudeau was modest in the extreme, always seeking to give someone else the credit; and not the credit only but the cash too, when there was any. In getting money for his charitable schemes he was a persistent and successful beggar; but the idea of making money for himself seems to have been utterly repugnant to this great-souled physician and truly heroic man.

